# Passive Equalization Networks—Efficient Synthesis Approach for High-Speed Signal Integrity Characterization

**DOI:** 10.3390/s21041222

**Published:** 2021-02-09

**Authors:** Diana Brinaru

**Affiliations:** Faculty of Electronics, Telecommunications and Information Technology, University POLITEHNICA of Bucharest, RO-060042 Bucharest, Romania; diana@munde.pub.ro; Tel.: +40-740-522-061

**Keywords:** equalization, signal integrity, high-speed, open stub, synthesis

## Abstract

High data rates challenges and long traces from current state-of-the-art systems imply high attenuation. In the present article, we will present a detailed process of synthesis of equalizers, for choosing the correct one for a given application. The methods are based on scattering parameters applied on interconnections modeled as microstrip or stripline. Firstly, one may have an overview of types of equalizers, passive, active, and adaptive ones, and a detailed filter synthesis is applied in microwave systems having as start point the insertion loss of a given trace on a given substrate. Next, time domain analyses offer a better understanding of the performance of the interconnect, based on eye diagram inspection and the variation of waveforms with time. Finally, we will present results based on simulation of the equalizers network in a microstrip technology followed by discussions and conclusions. The study proposes to use equalizers in either the transmitter or receiver point, proposes a bridge equalizer with the cost of additional elements but improved constant input, output impedance, and also a new variant for single ended trace based on microwave resonator is proposed. Performance is demonstrated by results from simulations.

## 1. Introduction

The challenge to transmit speeds of tens of gigabit per second in current state-of-the-art systems comes with several aspects concerning transmission media. Both frequency and time domains allow characterization of how distortion of a waveform travelling along a trace, even when high-performance techniques and materials are used. Clearly, the investigation is beyond the trace geometry or type of dielectric material used. The reason is that we are unable to solve the issue of trace loss effect on signals for high lengths and high data rates. Compensation techniques are applied in this specific case in a manner to gain a flat response in frequency, low and high equally. The basics of passive equalization was elaborated in [1].

Signals must have a finite propagation time or delay in reaching the destination due to a finite velocity for a given length of the interconnection between transmitter and receiver. Bits will arrive at a different time, with an offset, than when were sent. One must determine the sample time at the receiver for correct reconstruction of the original signal. We will consider for transmitter a digital non-return to zero signal type (NRZ). Another signaling option for high speed is multi-level pulse amplitude modulation (PAM) scheme for optimized interconnects as in the chip-to-chip applications, or backplanes [2,3]. For these variants the length will vary from few centimeters to one meter that will need to support high enough data rates. In the past, increase of data rates came with increased performance of I/O circuits (input/output), complex coding and modulation schemes, and use of more complex equalization.

A lossy line with both phase and amplitude distortion will have height pulse reduction and dispersion so the pulse smears outside of its assigned bit boundaries. Low frequency energy present in a transmission line will interfere with highly attenuated energy from the high frequency domain causing intersymbol interference (ISI).

In practice, it has been demonstrated that for better synthesis of a device, networks with equalizer (EQ) are positioned in the test fixture as presented in [4]. Jitter histogram, time, and frequency characteristics are more accurate in this case. As the data rate increases, the intersymbol interference effects on the jitter increase as well.

A line that does not preserve the proper amplitude and phase relations between harmonics will yield to distorted pulses. Because line losses are in direct proportion with the length of the line, the distortion will grow with the line’s length. 

Additionally, the characteristics of the lossy line of a lowpass filter, bandwidth limitation, and length dependency of the insertion loss (IL) result in amplitude attenuation and altering the phase relationship between harmonics. Since most digital signals have high edge rates, they will combine odd harmonics of the fundamental frequency. The transmission line’s loss is given by dielectric, conductor, and radiation characteristics, but for the present work the significant ones are the dielectric loss and skin depth due to conductor loss [5]. Several references provide analytical solutions to compute losses in a transmission line designed in a certain technology [5] but also software tools [6] are highly suitable to evaluate properly, accurately, the loss profile to be frequency dependent if algorithms are developed for materials parameter. The width of the line is a factor that influences the losses, as skin effect is inverse proportional to the width’s trace.

In the time domain, the effect of ISI is summarized as spread of the pulses travelling along the line with bandwidth limitation and in worst-case scenario it will smear into the neighboring time slots, leading to distortion of a bit within a symbol due to interference caused by pre- or post- bit from the transmitted stream.

Another cause of ISI is the residual energy caused in the line by signal reflections due to impedance mismatch, not at the ends where it can be controlled via internal impedance at the ports, but along the transmission line.

The present paper is focused on evaluation of interconnects in terms of previously presented aspects and different equalization schemes are applied to prevent ISI degradation evaluated via time and frequency domain analysis, based on simulation results. First, in Section 2, materials are selected for substrate, a certain technology is associated to the transmission link, distributed parameters are associated to model the interconnect, several properties of the transmitted signals on traces affected by higher and higher data rates and by increased length so increased attenuation, followed by general aspects of the EQ. In the same section particularities of passive, active, and adaptive EQs are then highlighted and compared in terms of functionality and complexity. Section 3 presents results of the passive EQs starting with a synthesis of the lumped elements based schemes and distributed ones using microstrip resonant structures. For the first part of this section, the synthesis process starts from the insertion loss of the analyzed link with a derivative resistor-capacitor (RC), a series of resistor-inductor (RL), or a combination of the two in a resistor-inductor-capacitor (RLC)—based EQ are first evaluated and in the end a bridge T filter is proposed for constant input and output impedance optimization function. For the second part, microstrip-based EQs, open stub structure will make the high-pass effect of EQ rise earlier and two variants of this model are implemented with a new schematic elaborated. The final section of conclusions summarize the synthesis presented in this paper.

## 2. Materials and Methods

**Figure 1:**
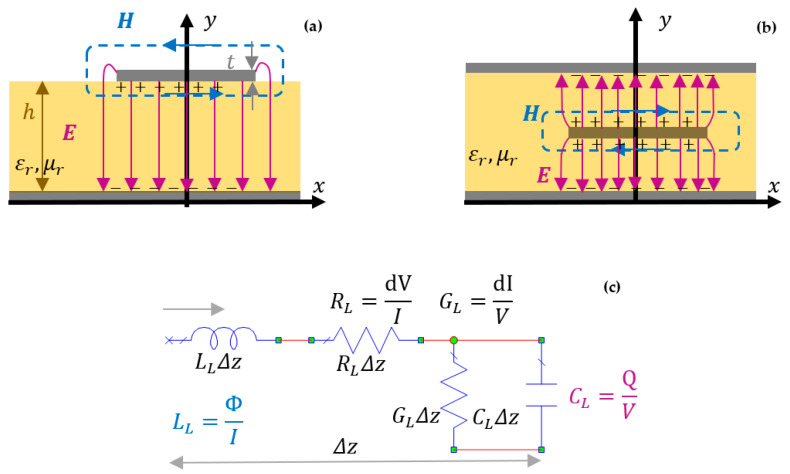
Transversal sections illustrating the E and H field intensities lines: (**a**) microstrip, (**b**) stripline, and (**c**) equivalent circuit for a very small line length Δz≪λ.

Since energy is conserved in a system, a part of the transmitted power goes into the trace before it reaches the load. The energy is given by electric and magnetic fields and can be modeled by inductor and capacitance per unit length as it is illustrated in Figure 1a–c. Typical technologies applied in interconnects analyses are microstrip (Figure 1a) and striplines (Figure 1b) for top or bottom traces or internal layers in a multilayer printed circuit board (PCB). Signals are always pairs of current–current flows in one terminal and flows out the other one. On these interconnects, high data rates are used in order to get bigger bandwidth, meanwhile keeping as much as possible a compact size of a digital or analog communication system. 

The particularities of high versus low frequency signals focus on distortion at high frequencies such as ground bounce, ringing, variation of propagation time, crosstalk, balanced, or unbalanced interconnects that are easily framed in frequency domain or time domain. For a given type of technology, the equivalent circuit with per unit length parameters for a very short length comparing to the wavelength, Δz≪λ, is represented in Figure 1c.

### 2.1. Typical Characteristics of Signals on Interconnections

**Figure 2:**
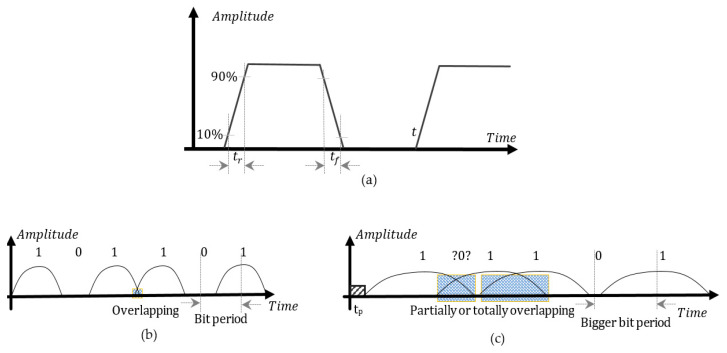
Pulse characteristics.

The input data at the interconnection near end is a sequence of bits introduced as file associated to the ARBS element (arbitrary, user-specified bit sequence) as arbitrary 0 s and 1 s for binary signaling condition. The sequence highlights the difference in propagation of high-speed data rate signals versus time variation. In Figure 2a, typical representation of pulse is presented to explain the main parameters that are setup in the design. 

As different harmonics of a given signal travel down a transmission line with different velocities and arrive at the end point with different propagation times, another aspect that can be deduced is the dispersion of the signal. A delay can be associated to a phase shift as harmonics are out of phase with one another.

Lossy line will make the pulse with altering amplitude and phase distortion have decreased amplitude, rounded edges, and its base will widen. The wider the base the smearer into adjacent bits, the bigger the energy spread of current bit.

**Figure 3:**
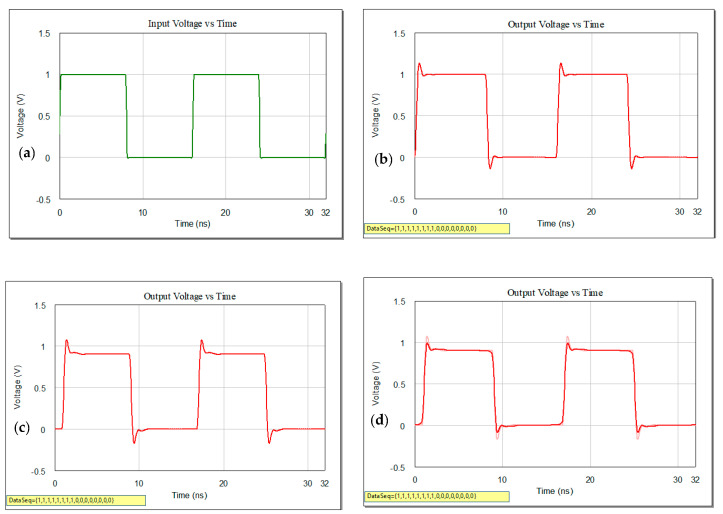

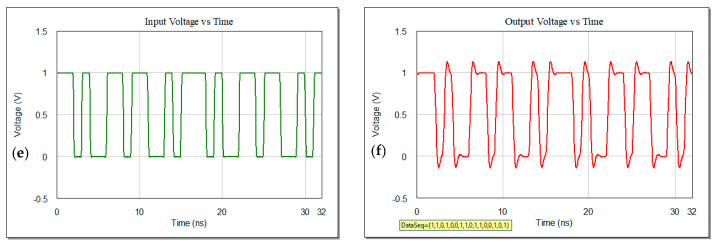
Voltage as variation in time: (**a**) at the input of the interconnection for back-to-back same polarity and (**b**–**d**) output of a microstrip line; (**e**) input voltage for high-speed data rate and (**f**) output voltage at the far end of microstrip line.

In Figure 3 on the left-hand side, a series of 1 s followed by a series of 0 s (in green) at the input of the line, with no rise and fall times and 1 V for the amplitude, will show increase overshoot and undershoot at the output of the line, as it results from the representation on the right-hand side (in red) (representation in a and b). Only changing the pattern in the data stream and alternate 1 and 0, the output waveform will evidentiate overshoot and undershoot similar as in previous case, but worth mentioning that a string of back-to-back 1 s reaches the receiver with higher amplitude than does the single 1 pulse. 

The lowpass characteristic of a lossy line, that includes losses in both dielectric and conductor materials, distorts the pulse shape. The pulse will be rounded as successive harmonics are eliminated given the distortion phenomena and the pulse will be attenuated with the increase of the line length or the increase of the losses. As the data stream contains pulses of various widths we will have gradually attenuated and phase shifts on the harmonics. Phase distortion in the pulse will give no longer flat-topped and bottomed and shows peaks as the improper phasing causes the harmonics to combine inadequately. If only amplitude distortion is present, the lossy line attenuates each harmonic differently. The effect of the improper combination of the amplitudes results in an output waveform with severe rounding of the corners, eliminated sharp edges, smoothed pulse with lower overall amplitude as it can be seen in Figure 3d with a zoom in on the smoothed edge. Another very important aspect that should also be mentioned is the spread of the pulse outside of the region occupied by the undistorted pulse.

Degradation of the signal is severe if the line cannot properly maintain the amplitude and phase distortions. The effect of a bandwidth-limited channel as a transmission line has on signal propagation is explained in next sections and in extenso in other texts [7,8].

**Figure 4:**
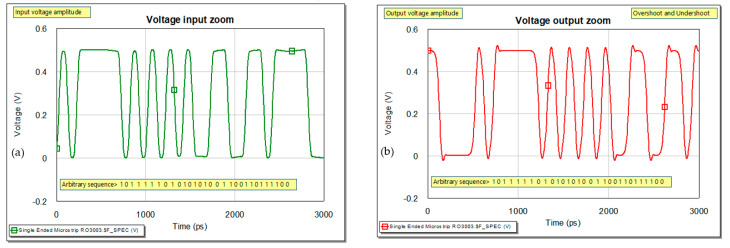
Voltage as variation in time: (**a**) waveform with rise and fall times of the pulses and (**b**) waveform at the output of the interconnect.

Following, we present the steps for matching at f=10 GHz the ports for a given substrate. RO3003 is used for the analyses. Details for the substrate are as follows: dielectric constant $\varepsilon_{r}=3$, loss tangent tanδ=0.001, substrate thickness h=1.524 mm, conductor thickness t=17.5 μm. [9] The waveforms at the input and output of a microstrip line for RO3003 are represented in Figure 4.

**Figure 5:**
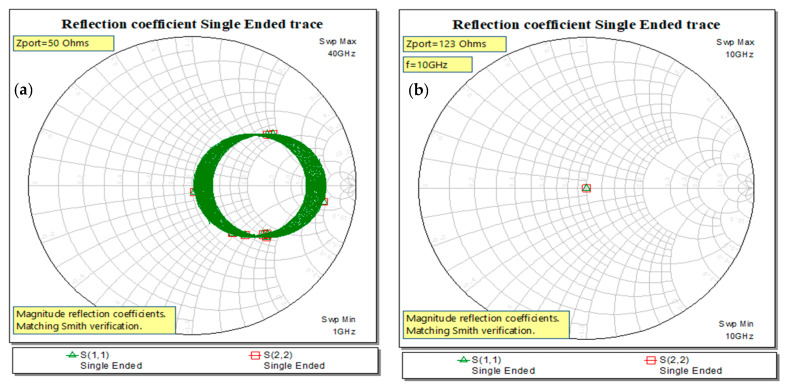
Matching the ports–Smith chart verification upon synthesis of microstrip line: (**a**) unmatched and (**b**) matched ports after synthesis for microstrip width, substrate and conductor definitions, simulation at 10 GHz, single point.

For matching the circuit, the Smith diagram is used below starting with mismatch in Figure 5a, using TxLine synthesis tool from Microwave Office (MWO) [6], the synthesis tool for different technologies included in MWO [6]. In Figure 5b, the progressive wave is obtained as the magnitude of the return loss is in the center of the Smith chart at 10 GHz, or, based on Nyquist rate frequency, the data rate is 20 Gbit/s.

**Figure 6:**
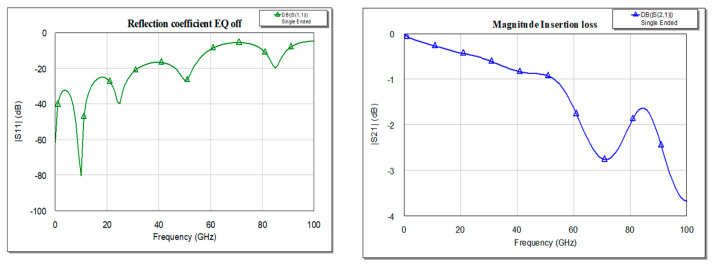
S parameters magnitude with frequency variation: (**a**) return loss and (**b**) insertion loss.

To determine the trace transfer function, below we represent the frequency variation of the insertion loss (IL). The frequency characteristics based on IL are similar to a low pass filter as can be deduced from Figure 6b. For the 10 GHz frequency selected to synthesize the circuit, the reflection loss presents general minimum of −80 dB (for a step in simulation process of 0.01 GHz) for the impedance port in Figure 6a.

### 2.2. General Aspects on EQ

**Figure 7:**
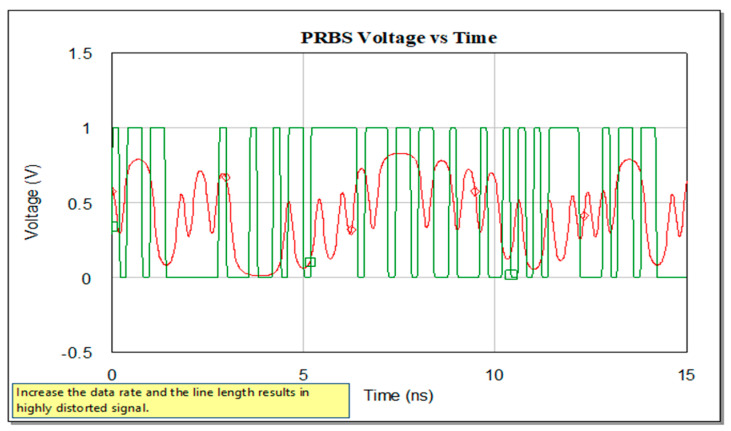
Voltage variation: at the input of the line, in green, and at the output of a long highly lossy line with high data rate transmission.

If the system implies a high data rate and long interconnections, the waveform at the input and output of the trace will be similar with the ones represented in the below figure. A received stream as in Figure 7, represented in red of the transmitted data stream implies definitely a solution to reconstruct the initial signal. A good solution is an EQ.

An EQ is a network designed with lumped elements or distributed ones, as transmission lines, active elements, or adaptive system, placed at one of the two ends of a trace or in both. Considering the generator at one end, the load at the far end, the current produced by the generator in the load should have a frequency variation that will compensate the insertion loss of the trace due to the dielectric and conductor losses that tend to increase with the increase of frequency. The EQ has to amplify the signal to count the attenuation at each low and high frequencies domains to avoid ISI. In other words, the EQ has to amplify high frequency oscillated wave or to reduce the signal magnitude at the low frequencies wave so the EQ will compensate and balance the two frequency domains, high and low. Given the components or function of the EQ, we shall have different variants:

- Active
- Passive
- Adaptive

Whether the components used in the EQ are active or passive ones, the EQ are commonly sorted as active EQ or passive EQ. The characteristic of EQ in this case should be similar to a high pass filter function. In the next section, passive EQ are analyzed and implemented in MWO.

### 2.3. Passive Equalization

The mathematical expression relates the transfer function of the EQ as the inverse of the line’s transfer function as in the relation [2]:

(1) $$H_{\mathrm{EQ}}\left(f\right)=H_{\mathrm{line}}^{-1}\left(f\right),$$

or, if the transfer function of the line is known actually as the IL of the line or the $S_{21}$ scattering parameter, the EQ transfer function is defined by the relation:

(2) $$H_{\mathrm{EQ}}\left(f\right)=H_{\mathrm{comp}}\left(f\right)/H_{\mathrm{line}}\left(f\right),$$

**Figure 8:**
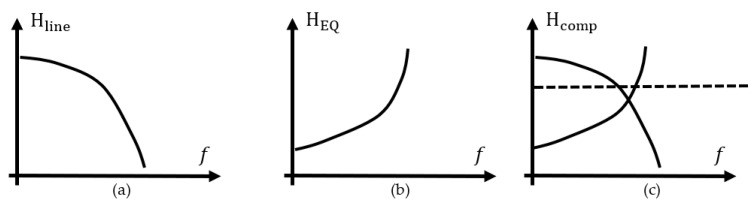
Transfer functions: (**a**) lossy line (**b**) EQ and (**c**) compensated line (dashed trace).

The meaning of $H_{\mathrm{comp}}\left(f\right)$ is the overall transfer function of the system after EQ is part of transmitter, channel, or receiver system and is represented at theoretical level in Figure 8c. In the frequency domain, $H_{\mathrm{comp}}\left(f\right)$ is obtained by multiplication of $H_{\mathrm{EQ}}\left(f\right)$ and $H_{\mathrm{line}}\left(f\right)$ and in time domain, as convolution of associated time domain impulse functions for line and EQ. Amplitude and phase distortion in the new circuit with the EQ added needs to reverse the effects of the interconnect to a perfectly original signal form. This is the ideal case, but in practice, there is no perfect EQ and throughout of the following work, we will demonstrate that there are various implementations, discrete and distributed, that compensate the line’s imperfections for multi-gigabits per second signaling systems. 

Points of insertion of the EQ are either at the receiver end, post-emphasis, or to the transmitter, pre-emphasis. In the first case, the driver’s output current is increased over the nominal value when the bit is transitioning [2]. The analysis done in the pre-emphasis block is based on single bit or multiple bits from the data stream. Firstly, the current is reduced by a predetermined amount only when at transitions 0 to 1 or 1 to 0. For bits with same polarity, the nominal current value is lower. The high-frequency transitions are, in conclusion, pre-emphasis relative to the back-to-back same polarity bits (series of 1 s or 0 s) thus high frequency-like domain is transmitted with more energy than low frequency domain [2].

**Figure 9:**
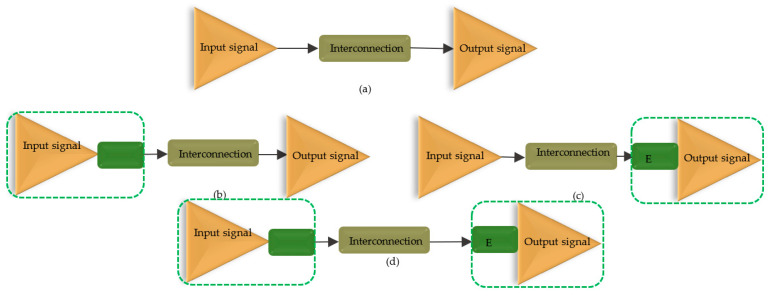
Block diagram: (**a**) trace without EQ, (**b**) active pre−emphasis, (**c**) active post−emphasis, and (**d**) pre- and post-emphases.

Based on the location of the emphasis system in the chain we shall have as depicted in the below Figure 9:

- Pre-emphasis
- Post-emphasis
- Pre- and post-emphasis

Often, high-speed designs use the eye diagram to evaluate system performance. The construction of the eye diagram assumes slices into sections of the time domain signal waveform followed by overlaying process [5,10]. These sections are considered a small number of symbols in length. Folding the input signal on a time segment of length equal to the symbol period allows voltage amplitude representation on the vertical axis, while on the horizontal is the time variation that includes typically one or two symbols. Signal distortions are easy to be demonstrated on the eye diagrams [10]. The closer the eye, the more distortion we have on the signal and larger eye opening implies more margin to the voltage and timing requirements. 

**Figure 10:**
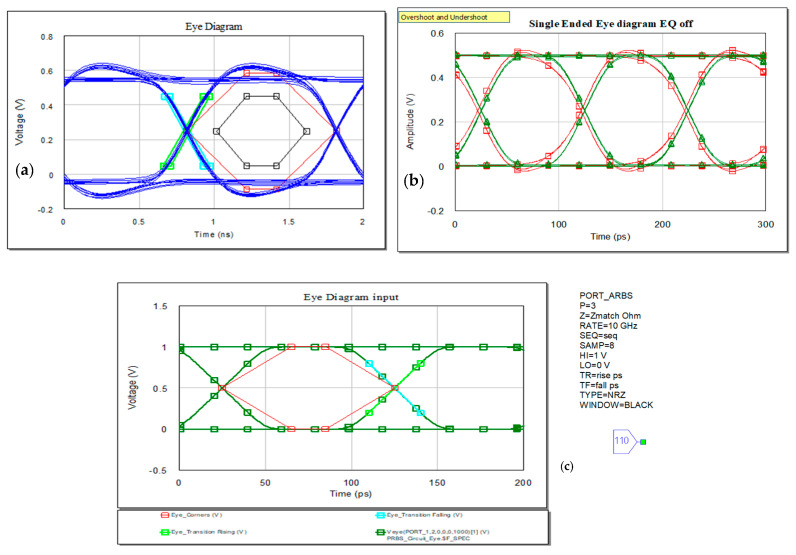
Time domain analysis: (**a**) eye diagram typical representation with mask representation (in black), and corners marked (in red), (**b**) eye diagram for the specific arbitrary random sequence applied and determined at the output of the trace, and (**c**) eye diagram and metrics at initial point of transmission.

There are several metrics that can be achieved by representation of the eye: a mask can evidentiate unwanted crossings, corners, transitions, and also gives a feedback of the jitter measurements. The height of the eye should be large enough to ensure the voltage thresholds for high and low levels assuming additive noise additionally degrades the system performance. Furthermore, the eye should be wide enough to provide adequate timing margins for the receiver. The variations in voltage and timing are noise and jitter and using these metrics one can calculate the system’s budget. The mask mentioned earlier in this section represents a forbidden region that should not be crossed over imposed in the eye diagram representation (in black represented in Figure 10a). It is a method to find the minimum voltage height and time width at the receiver end in a signaling systems affecting the amplitude and phase due to loss and mismatch. It is based on peak distortion analysis method.

Crossing the mask’s limits, we can also estimate the probability of receiving erronate bits, so we measure the bit error rate (BER) defined as the ratio of the erroneous receive bits over the total number of transmitted bits in a sufficiently long time interval [5].

Continuous time linear EQ has analog elements and does not require digital devices for implementation. Schematics based on passive components have parallel and/or series inductors, capacitors, and resistors connected to the line at its ends based on EQ behavior.

### 2.4. Active Equalization

EQ can also be designed with the use of active elements as amplifiers to gain improvement. The active EQ usually consists of passive EQ and additionally an amplifier is used to enhance the signal’s level in the entire frequency domain, high and low. The main disadvantage of this type of active EQ is the overall cost, as more active and passive circuit components are used, but also the increased area occupied by EQ will result in large dimensions of the system. This method is often done by using a split-path approach [11]. The input is split in two paths: one that has unity gain and another is to be high frequency boost. At the end, the two signals are summed into a new equalized input signal into the lossy line [2]. 

### 2.5. Adaptive Equalization

Systems that are more complex employ training sequences for transmitter’s compensation settings for tuning to the best performance. After a given period, the adjustment is remade. The transmitter and receiver are set to automatically adapt to the changing conditions of the interconnect. For this purpose, the scheme requires that the transmitter and receiver communicate with each other in a known and predictable way [5]. At intermediate data rates, the EQ coefficients are often set based on the average characteristic of the line. The design of the link introduces dependencies on technology corners that need to be compensated by the EQ via calibration and adaptive techniques of the EQ parameters for the actual channel. Usually variants of least mean square (LMS) algorithm are implemented. For rates in orders of Gb/s, Sign-Sign LMS is often the only option due to very short time needed to determine the EQ parameters. Based on probabilistic considerations applied to the sampled pulse response, the LMS convergence methodologies estimate the evolution in EQ structure [12]. This approach is based on permutation matrix to take into account the probability of the transmitted sequences and find adjustments to the LMS parameters. Evaluation in terms of cost, constraints, implementation, power, performance of CTLE (Continuous Time Linear Equalizer), FFE (Feed-Forward Equalizer), DFE (Decision Feedback Equalizer), or FEC (Forward Error Correction) for high data rates in [12] and [13] conclude that while DFE can reduce channel ISI with short tap length, FFE is needed to compensate pre-cursor ISI and compensate the power-voltage variations of transceivers, and also fits in DSP (Digital Signal Processing) implementation.

The benefit of adaptive equalization is the ability to adjust lengths and data rates. The main disadvantages are an increased complexity and power consumption. 

## 3. Results

Some of the EQs presented variants in previous sections are implemented in MWO Schematic. The substrate’s parameters are presented in Table 1.

Data stream PORT_ARBS was used with declaration of variable called DataSeq as in relation (3) in the simulation project as global variable and for PORT_ARBS the symbol sequence is assign to DataSeq.

**Figure 11:**
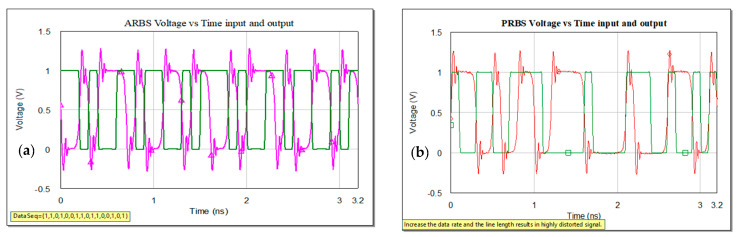
Voltage variation with time: (**a**) input signal arbitrary, user specified bit sequence (ARBS) source at 10 GHz (green) and output after 10 cm microstrip line (pink) and (**b**) input signal pseudorandom bit sequence (PRBS) source at 10 GHz (green) and output after 10 cm microstrip line (red).

(3) DataSeq={1,1,0,1,0,0,1,1,0,1,1,0,0,1,0,1},

The data input stream is defined as series of 1 s and 0 s as represented in Figure 11. Below are represented variants of bits sources, ARBS and PRBS, pseudorandom bit sequence, at the input of a microstrip line with the substrate RO3003. Symbol rate is set in both sources at 10 GHz, samples per symbols at 16, and in the case of PRBS, 64 is the number of symbols and 1 bit in symbol via BITW, bit width.

(4) $$\begin{array}{c} \mathrm{Rates}=\left\{1,2.5,5,10,20,30,40\right\}\\ \mathrm{ModFreq}=\mathrm{Rates}\;[4] \end{array}$$

Similar effects can be observed in both representations. The signal at the output of the microstrip line is highly distorted, both in amplitude and in phase, increased overshoot and undershoot due to frequency transmitted. 

**Figure 12:**
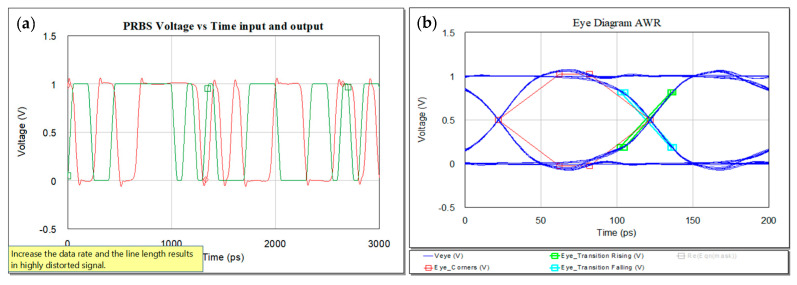
Voltage variation with time for 50 ps rise and fall times (**a**) and eye diagram associated at the output of the microstrip line (**b**).

For 50 ps rise and fall times, the signal is represented in Figure 12 with the input and output waveform in (a) and the eye diagram at the output of the microstrip line.

### 3.1. Passive EQ-Lumped Elements, Synthesis

Passive EQ does not amplify any components of the signal, low or high frequencies that pass through it but rather attenuates the low frequency signal components. 

A method to determine the filter’s elements [14] starts from insertion loss representation applying filters’ synthesis theory before applying any EQ scheme. At the cut-off frequency, $f_{c}$, the EQ’s frequency, determined from the dB IL of the trace, the value of the attenuation is half of the maximum value. In the frequency domain selected of [0, 40] GHz, the maximum is ${\mathrm{IL}|}_{\max}={\mathrm{IL}|}_{40\;\mathrm{GHz}}=-0.82\;\mathrm{dB}$ so $f_{c}≅20\;\mathrm{GHz}$. Applying the theory of filters’ synthesis, the IL [dB] at a given frequency f is offered here without proof [14] by the relation:

(5) $$\mathrm{IL}\left(f\right)=10\log\left[1+\frac{K^{2}-1}{1+K{\left(\frac{f}{f_{c}}\right)}^{2}}\right],$$

where factor K determines the filter’s IL, or the amount of attenuation provided, measured at frequency $f_{c}$, but is also a measure of the flatness of the transfer function, that should be equal with the inverse transfer function of the trace. From relation (5), it can be deduced that the higher the K, the flatter the frequency response. Typically, K∈(1,5) otherwise a value greater than five causes a flatness of the response in frequency domain and the IL will increase [2]. 

**Figure 13:**
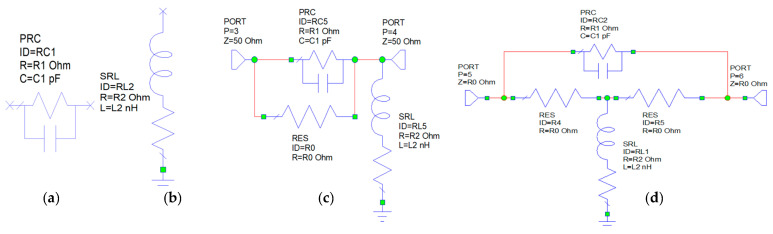
Types of passive EQs: (**a**) RC, (**b**) RL, (**c**) and (**d**) composed RC and RL in bridged variants.

Next, value of the factor is determined for the present case for f=10 GHz. From the representation of IL, we deduce that IL(10 GHz)=−0.25 dB. From this, K is determined to have two values, only one corresponding to a positive value in the interval (1, 5), and that is K=1.145. There are several architectures to be considered so that the EQ performs as a high pass filter [14]. For passive type, EQ variants will include RC derivative shunt of a resistor and a capacitor connected as in Figure 13a or RL series of a resistor and an inductor connected as in Figure 13b or combination of the two, or bridge type EQs as in the below representation from Figure 13. 

From K coefficient deduced at the working frequency, the cut-off inductance and capacitance [14] are:

(6) $$L_{c}=\frac{R_{0}}{2{\pi f}_{c}}\;C_{c}=\frac{1}{2{\pi f}_{c}R_{0}},$$

where $f_{c}$ is determined from (7):

(7) $$f_{c}=\frac{1}{2\sqrt{L_{c}C_{c}}}={\frac{1}{2}\mathrm{IL}|}_{\left[\mathrm{dB}\right]},$$

and $R_{0}$ is the EQ impedance, in this case $R_{0}=123.65\;\Omega$ determined with TxLine. Computing the values for $L_{c}$ and $C_{c}$ results 0.983 nH and 0.0643 pF so that $L_{1}$ and $C_{2}$ for the EQ components are:

(8) $$L_{2}=L_{c}\frac{\sqrt{K}}{K-1}=7.25\;\mathrm{nH},\;C_{1}=C_{c}\frac{\sqrt{K}}{K-1}=0.474\;\mathrm{pF},$$

and the resistive elements are:

(9) $$R_{1}=R_{0}\left(K-1\right)=17.92\;\Omega ,$$

The computed values of the elements for the EQ networks were used in the MWO [6] and results are presented in the next subsections.

Another variant as presented in the Figure 13d is the constant input and constant output impedance bridge T. The values of the components are the same deduced in relations (8) and (9). The cost of this EQ is increased due to additional resistive parts. 

The bridged EQ is also implemented in MWO and presented in the next subsections. Other EQ networks were implemented in MWO [6] but the performance was limited both in time and frequency domains.

#### 3.1.1. RC EQ

**Figure 14:**
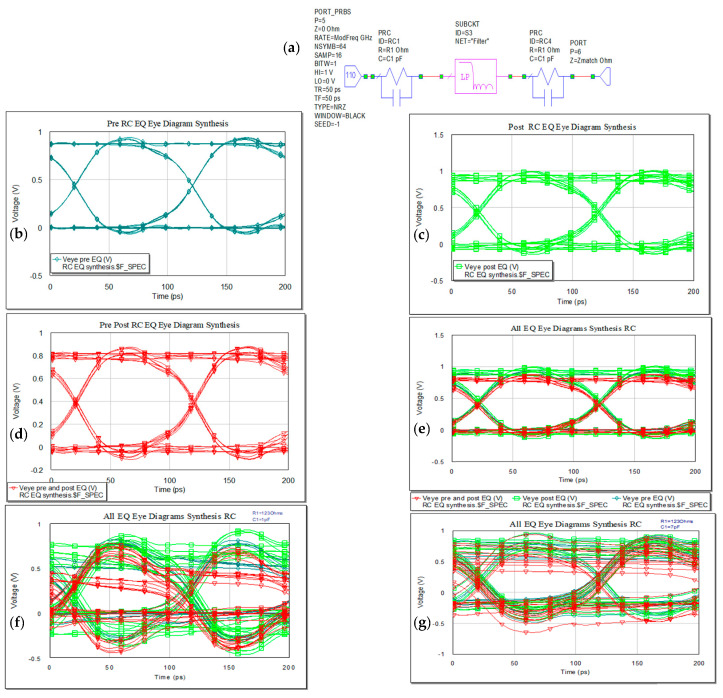
RC EQ: (**a**) schematic, (**b**) pre (**c**) post-, (**d**) pre- and post-, and, (**e**) overlaid results obtained from synthesis process. (**f**,**g**) result from selection of R_1_ and C_1_ values.

A passive EQ with RC derivative structure is next integrated in the circuit. Time and frequency domain analyses present better performance for connecting the EQ prior to the microstrip trace. The values of the EQ can be determined via an optimization process in MWO tuning; accordingly, the R_1_ and C_1_ values or synthesis process can be applied as it was presented in earlier subsections. Better results were obtained via synthesis but the graphical results are presented in Figure 14f,g for keeping R_1_ = Z_match_ of the port, constant value, and C_1_ value varying from 1 pF to 7 pF from tuning process. The eye in this case is close for smaller value of the capacitor and also the reflections are higher in the case of small values of C_1_.

The high level peak is at 941 mV. Connecting the EQ at both ends of the line, the eye diagram resulted is closer comparing to the other two variants and also jitter has a higher value in this case. Better results were obtained via synthesis process.

**Figure 15:**
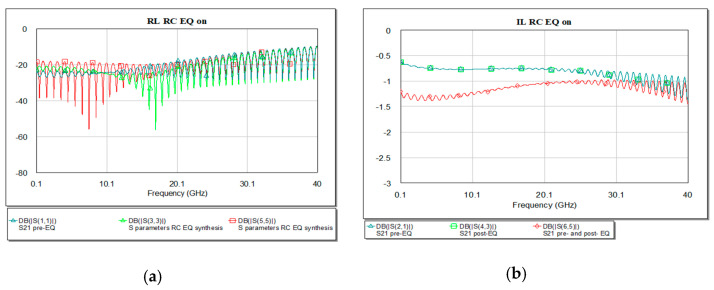
Insertion (**a**) and return loss (**b**) in the RC EQ: pre- (light green), post- (dark green), pre- and post- (red), and overlaid for the synthesis results.

Reflection loss presents good response for combination of pre- and post-EQ placed at both ends of the microstrip line at the working frequency as it is represented in Figure 15.

#### 3.1.2. RL EQ

**Figure 16:**
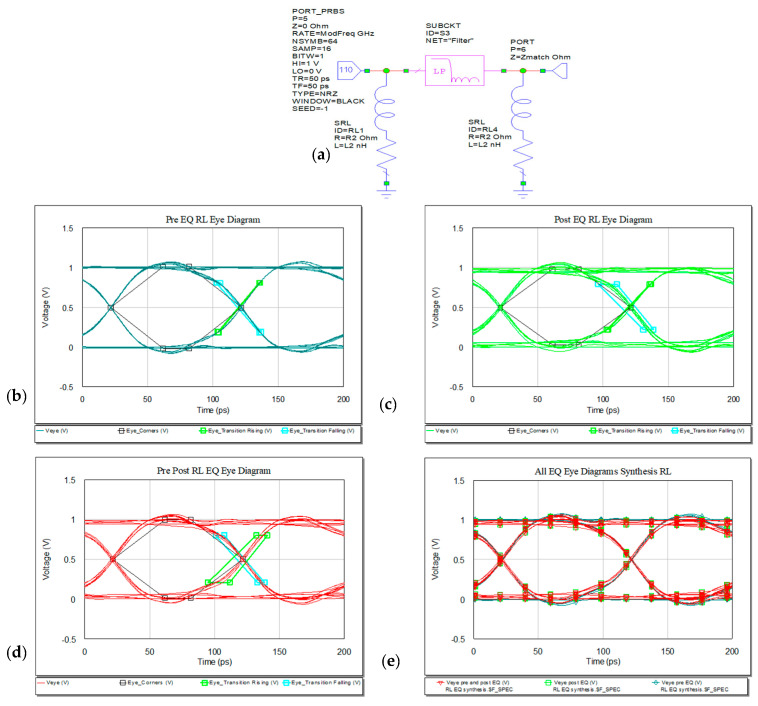
RL EQ: (**a**) circuit, (**b**) pre-, (**c**) post-, (**d**) pre- and post- and (**e**) overlaid.

A passive EQ with RL series connected in parallel to the line is next analyzed both in frequency and time domains. The values can be either determined via optimization process in MWO or can be computed in the synthesis process. The resistor is used mainly for matching purposes at the ports and the series inductor has to compensate the high frequency loss. The high level peak is at 1.066 V, so the circuit still has overshoots and undershoots in the waveform representation in Figure 16. Connecting the EQ at both ends of the line, the resulting eye diagram has a smaller peak compared to the other two variants (pre- or post-EQ for RL type).

**Figure 17:**
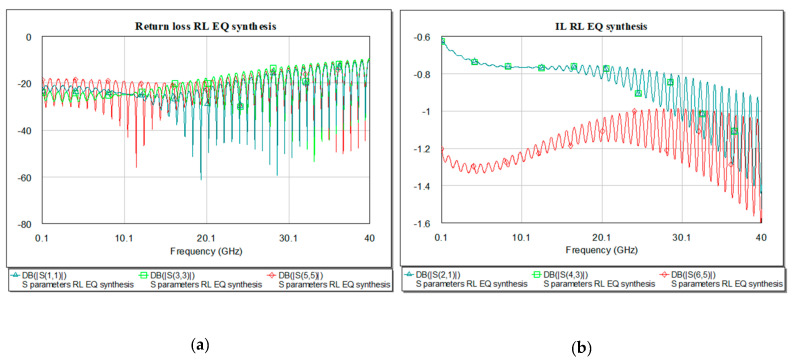
Insertion (**a**) and return loss (**b**) in the RC EQ: pre- (light green), post- (dark green), pre- and post- (red), and overlaid.

The return and insertion loss for the present circuit shows in Figure 17 a resonance at 10 GHz of −19.08 dB.

Reflection loss presents good response for combination of pre− and post−EQ placed at both ends of the microstrip line as in Figure 17.

#### 3.1.3. RLC EQ

Implementing RLC EQ results in attenuation of the transmitted signal as the peaks no longer pass the thresholds. The waveforms and eye diagrams for pre-, post-, and pre- and post- microstrip line. The SUBCKT “Filter” is the actual microstrip line. The variant of either pre- and post- EQ gives less attenuated amplitude. The maximum obtained in pre-EQ is 924.26 mV and in post-EQ the maximum is 934.53 mV and in case of pre- and post- the maximum amplitude is 803.41 mV. 

**Figure 18:**
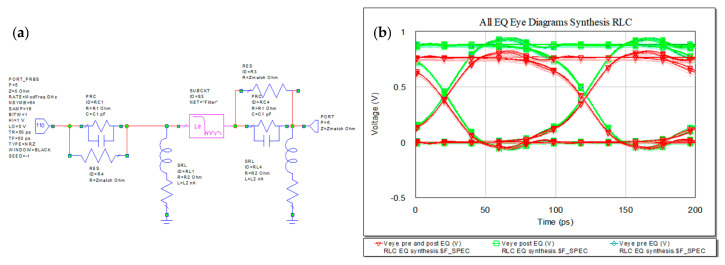
Variant of RLC EQ (**a**) circuit schematic and (**b**) eye diagrams with $Z_{\mathrm{match}}$ connected to the RC.

**Figure 19:**
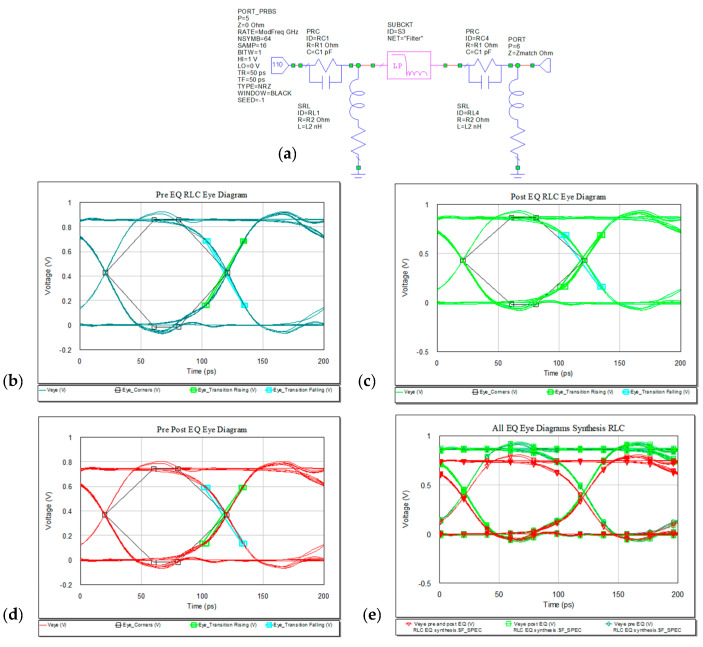
RLC EQ: (**a**) circuit, (**b**) pre-, (**c**) post-, (**d**) pre- and post-, and (**e**) overlaid.

Another variant of EQ filter is presented in the below Figure 18. The new EQ filter is slightly improving the eye opening for the case of implementation as presented in (a) comparing to the variant in Figure 19a. In Figure 18b, with EQ connected in front of the link and Zmatch connected derivative to the previous RC structure, both eye diagrams are represented freezing in the graph, the results given by Figure 19a output circuit.

**Figure 20:**
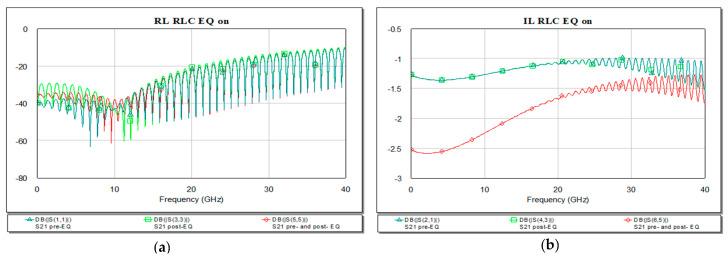
Insertion (**a**) and return loss (**b**) in the RLC EQ: pre- (light green), post- (dark green), pre- and post- (red) and overlaid.

In frequency domain, we can see the RL and IL after introducing the EQ in the system. The IL will need to have a more flat curve due to high frequency compensation. For the present design the RL and IL are represented in Figure 20 below. The best match is obtained using the RLC EQ at the both ends of the microstrip, −61 dB at 9.59 GHz.

**Figure 21:**
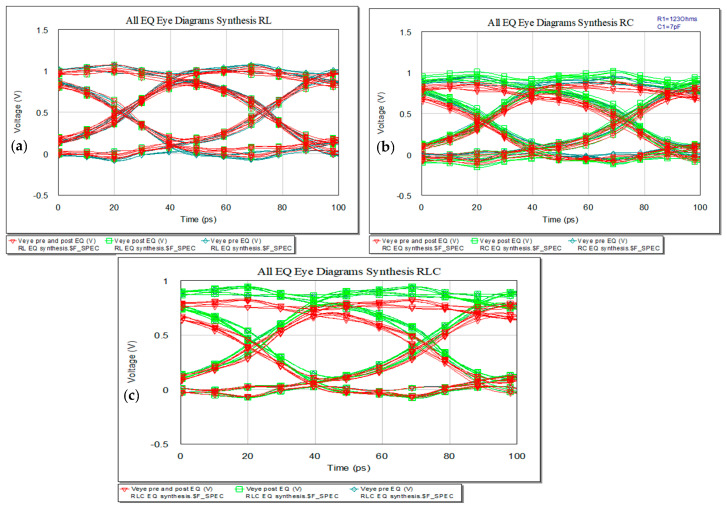
Eye diagrams for increased data rate: (**a**) RL EQ, (**b**) RC EQ, and (**c**) RLC EQ.

Increasing the data rate to 20 Gbps, the eye diagrams still maintain proper openings as represented in Figure 21.

#### 3.1.4. Bridge T EQ

**Figure 22:**
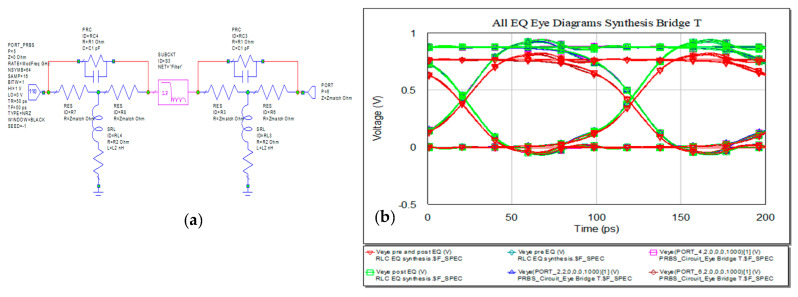
Variant of Bridge T EQ (**a**) circuit schematic and (**b**) eye diagrams in pre-, post-, and pre- and post- line for Bridge T and RLC EQ modified.

For constant input and output impedance, another solution for EQ network is the Bridge T network as represented in Figure 22. As explained in the beginning of this section at the synthesis for this type of networks, the values for inductors, capacitors and resistors results from the IL characteristic of the line.

**Figure 23:**
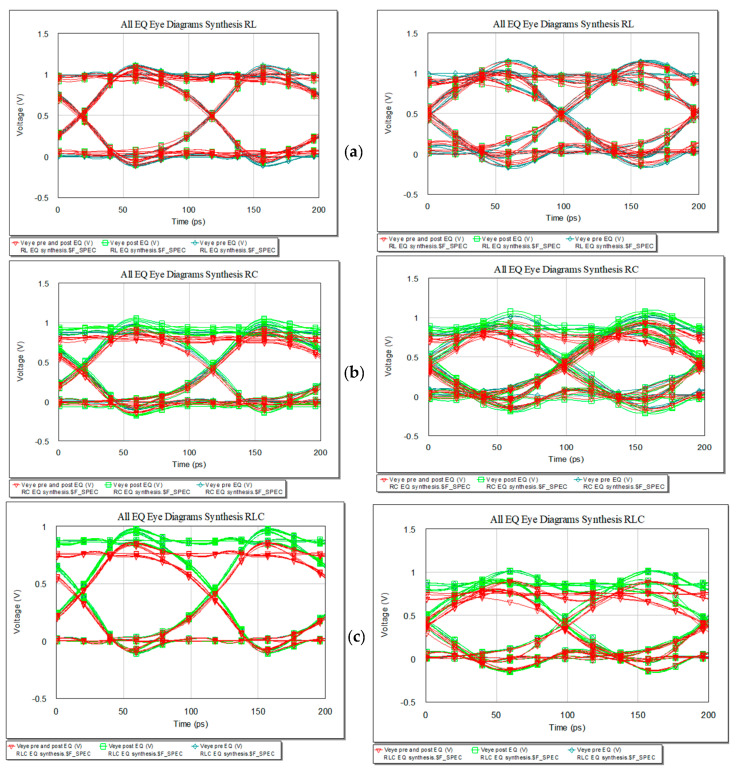
Eye diagrams for increased line length for RL (**a**), RC (**b**), and RLC bridge T variant (**c**) (from left to right): 20 cm and 50 cm.

If the length will increase, the IL will rise. Keeping the 10 Gbps data rate, the new eye diagrams for the three variants are presented in Figure 23 with Figure 23a for RL EQ, Figure 23b for RC EQ and Figure 23c for RLC EQ. The first column in representation of the length is set to 20 cm and the second one with a length of 50 cm. The eye diagrams still fit the height and width margins for 20 cm with overshoot and undershoot but highly exceeds the limits for 50 cm, still with clear open eye. 

The EQ cannot provide the function of equalization for the given IL at these lengths as distortions increase with the length of the interconnection. In this case, a new synthesis has to be done.

**Figure 24:**
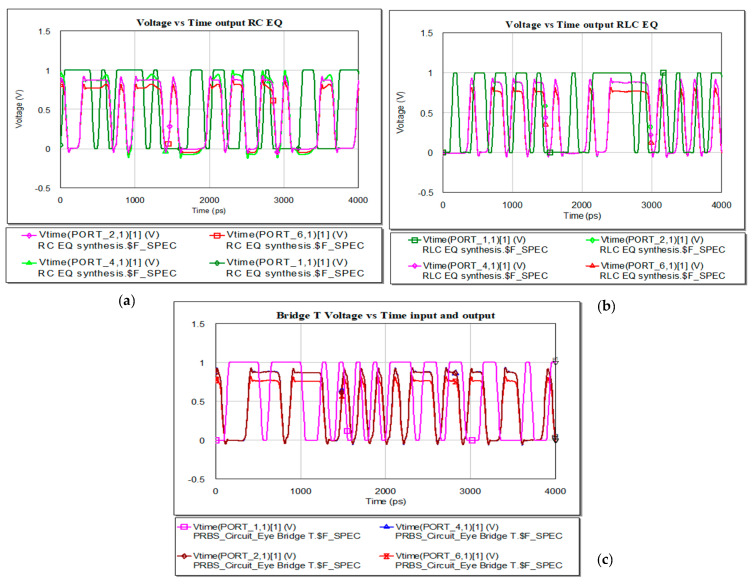
Waveforms at the input and output: (**a**) RC EQ (**b**) RLC EQ and (**c**) Bridge T RLC.

The waveform at the output of RC and RLC type of EQ are represented in Figure 24. In the case of RC EQ type implemented, only the pre−EQ variant will reach the high level but the undershoot exceed the low level. The peaks pass the low level as it is represented in the voltage variation in RLC EQ case. 

Based on the three variants implemented above one can select based on constraints applied to the own system. 

### 3.2. Passive EQ—Distributed Parameters

A solution to replace the lumped elements in the EQ is the use of transmission lines equivalent to the components. Some solutions were proposed [15,16,17,18,19], to include microstrip-based structures in the EQ form, due to size reduction in the mainline or wideband frequency response. Solutions for gaining EQ based on ceramic substrate with microstrip stepped impedance resonators also increase the bandwidth and input/output return loss as presented in [20]. If the resonant structure is replaced in the EQ with a composite right-/left-handed (CRLH) structure, a reduced size should be obtained as a shorter CRLH can realize the same resonant frequency compared to right-handed structure, in the traditional EQ [21].

**Figure 25:**
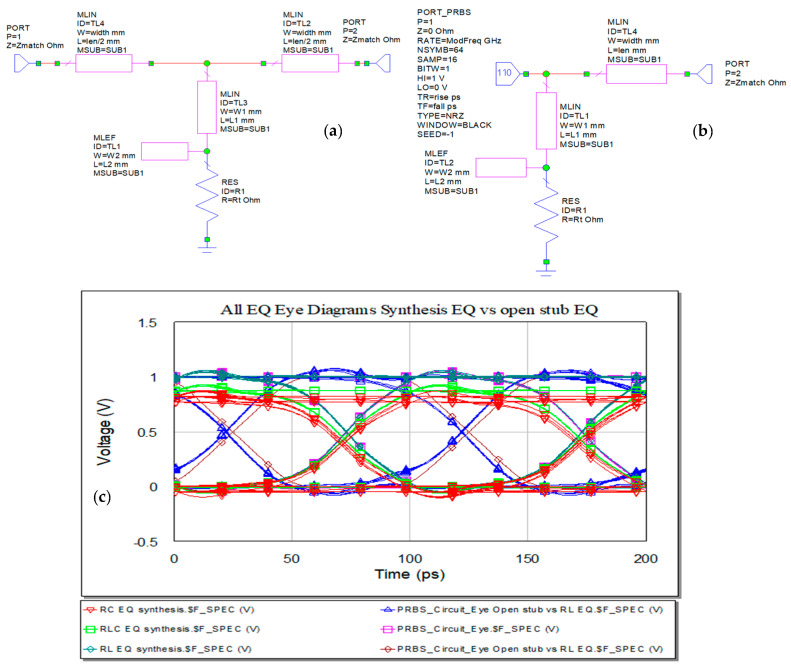
Open stub EQ: (**a**) splitted line, (**b**) pre-EQ with open stub, and (**c**) eye diagram—in blue is represented the eye diagram for the proposed EQ.

Solutions presented include an open stub in an RL EQ series structure [15]. The RL EQ placed in the middle of the microstrip line will perform the high-pass filtering. At low frequency, the inductor’s impedance has lower value comparing to the termination resistor, but its impedance will dominate at high frequency [22]. This method can greatly alleviate the frequency dependent loss, transform the IL into a flat curve, and though reduce the ISI. Before the series resistance of the RL network in [15], it is proposed to connect an open stub that will be equivalent to a LC-series resonator to shunt the equivalent reactance to the ground. The schematic is represented in Figure 25a. The open stub has to make the high-pass effect of EQ sharper, so the frequency response of the transmission line can rise earlier than in RL EQ [15].

A new variant of this design is proposed below in Figure 25b. The IL at 10 GHz is two times lower comparing to the individual trace and the eye diagram is represented in Figure 25c. Dimensions of the transmission lines that replace the LC resonator equivalent to the open stub can be deduced via synthesis process for the chosen working frequency. Another solution may be suited from an optimization process. The values for widths and lengths of the lines are in Table 2.

Fitting the input and output impedances of the circuit can further improve this design. The designed circuit has a return loss at 10 GHz of −53 dB, so better performance comparing to the purely lumped EQs. Furthermore, the EM analysis may give another approach and new perspectives for this network. 

## 4. Discussion and Conclusions

In terms of electrical perspectives, a PCB physical channel is characterized by frequency-dependent material losses, reflections from discontinuities as connector board interfaces and interferences from neighboring data paths. Attenuated and dispersed signal leads to ISI. Reflections are addressed in modern links with via back drilling, T-coil matching networks or optimized connector interfaces as presented in [10,17]. Conventional approaches to EQ design are based on time domain simulations along with experimental validation of results in an iterative way until specifications are met [10]. For a given frequency range, an EQ should fit the following rules: approximately flat transfer function, matched input and output, and low residual losses. An example of amplitude EQ is presented in [16] with resistor loaded, a resonant unit and the link represented through a transmission line. 

In the previous sections, we described the use of EQ in the signaling systems with in depth analyses on the passive EQ. The performance of the system is limited by ISI due to frequency-dependent IL of the line. The scope of EQ is to flatten the frequency response of the transmission system to increase the bandwidth of the line and to be able to increase the data rates. EQs may be included in the transmitter or receiver or split between the two.

Using the filters’ synthesis approach, we started the analysis from the IL expressed in dB of the given microstrip single ended line. The K coefficient was resolved and a series of EQ were synthesized as follows: derivative RC, series RL, combined of the first two variants for RLC EQ, bridged T RLC, and RL variants with open stub inserted to overcome the frequency-dependent behavior of the interconnect which were implemented in MWO. The EQ has to compensate for high frequency attenuation. Clearly, the EQ compensated for lossy line’s frequency behavior, making IL flatter across frequency domain. However, the flatness implies an overall attenuated amplitude of the signal. A practical EQ trade off the IL for flatness response.

The time domain simulation results show clear improvement on the data eye metrics. Before integration in the circuit of the EQ, overshoot and undershoot were observed in the waveform at the output of the interconnect due to fast rise and fall time of the simulated source and because the IL has no flat characteristic in frequency domain. In practice, a real driver is typically slower and the signal path’s loss is slightly higher so a small overshoot in simulation can be accepted. The new microstrip resonator structure proposed was evaluated by simulation means in time domain and evidentiated similar results with the existing model in [15], but with the advantage of positioning in front of the link. Future evaluation of the proposed EQ might show some limitations of the structure.

Passive EQs offer the advantage of improved performance with no additional power consumption. Moreover, we need to mention that this type of EQ fits easier integration into the silicon. However, they demand tighter control of the components values for typical digital applications. In addition, variation of the frequency response for passive EQ implies active circuitry that will tend to degrade the power benefit.

Further investigation is based on the last variant of distributed elements EQ implemented at the schematic level in previous section.

## Figures and Tables

**Table 1:** RO3003 substrate.

Relative Dielectric Constant (/)	Loss Tangent of Dielectric (/)	Dielectric Thickness (mm)	Conductor Thickness (μm)
3	0.001	1.524	17.5

**Table 2:** Dimensioning the open stub EQ.

Transmission Line Parameters	TL1	TL2
Length (mm)	5	4.43
Width (mm)	0.43	1.26

## Data Availability

The data presented in this study are available on request from the author.

## Abbreviations

The following abbreviations are used in this manuscript:

ARBS	Arbitrary Bit Sequence
AWR	Applied Wave Research
BER	Bit Error Rate
BITW	Bit Width
CRLH	Composite Right/Left-Handed
CTLE	Continuous Time Linear Equalizer
DFE	Decision Feedback Equalizer
DSP	Digital Signal Processing
EQ	Equalizer
FEC	Forward Error Correction
FFE	Feed-Forward Equalizer
I/O	Input/Output
IL	Insertion loss
ISI	Intersymbol interference
LMS	Least Mean Square
MWO	Microwave Office
NRZ	Non Return to Zero
PAM	Pulse Amplitude Modulation
PCB	Printed Circuit Board
PRBS	Pseudo Random Bit Sequence
PRC	Parallel RC
RC	Resistor Capacitor
RLC	Resistor inductor capacitor
S	Scattering
SI	Signal Integrity
SLR	Series LR
